# Lactulose-Induced Pneumatosis Intestinalis Causing Sudden Deterioration in a Patient With Advanced Hepatic Disease

**DOI:** 10.7759/cureus.78061

**Published:** 2025-01-27

**Authors:** Marielle Roberts-McDonald, Baker Edrees, Muhammad Ali Khalid, Rida Ghani, Lawrence Goldstein

**Affiliations:** 1 General Surgery, Internal Medicine, Ross University School of Medicine, Bridgetown, BRB; 2 Internal Medicine, Florida International University, Herbert Wertheim College of Medicine, Miami, USA; 3 Internal Medicine, American University of Antigua, St. John's, ATG; 4 Internal Medicine, Western Reserve Health Education, Warren, USA; 5 Pulmonary and Critical Care Medicine, Western Reserve Health Education, Cleveland, USA; 6 Pulmonary and Critical Care Medicine, Northeast Ohio Medical University, Rootstown, USA

**Keywords:** advanced hepatic disease, hyperammonemia-encephalopathy, lactulose, medical intensive care unit, pneumatosis intestinalis

## Abstract

Herein, we present a case of a 53-year-old male who developed pneumatosis intestinalis (PI) while being treated for hepatic encephalopathy with lactulose. Initially, the patient was administered lactulose, the standard treatment for lowering ammonia levels associated with hepatic encephalopathy; however, this approach unexpectedly resulted in the acute onset of severe PI, which persisted even after lactulose was stopped, further compromising the patient's health. Despite intensive management and the cessation of lactulose, the patient's condition rapidly declined, leading to a transition to palliative care and eventual death. Comparative studies have indicated that both PI and pneumoperitoneum can be triggered by lactulose therapy, with symptoms reversing upon the discontinuation of the medication. This case raises significant concerns regarding the use of lactulose in patients suffering from advanced hepatic disease and multiple comorbidities.

## Introduction

Pneumatosis intestinalis (PI) is a rare clinical condition that affects approximately 0.3% of the population, with 15% of cases attributed to primary causes and 85% to secondary causes [1]. This condition is characterized by free air and gas, with hydrogen constituting about 50% of the gas content [2], in the extraluminal space of the intestine. PI is predominantly localized to the small and large intestine within the gastrointestinal (GI) tract and may be associated with pneumoperitoneum and portal venous gas [2]. The clinical manifestation of PI can range widely, from an incidental radiologic finding to a severe, life-threatening emergency. 

Three primary theories elucidate the pathogenesis of PI: mechanical, pulmonic, and bacterial, each pointing towards the development of PI [1]. The mechanical theory posits that mucosal injury may arise from increased intraluminal pressure and gas in mesenteric vasculature, often resulting from surgical complications, endoscopies, or previous lung diseases [1]. The pulmonary theory explains how gas can transverse through mediastinal vessels, circulating caudally from the mediastinum into the retroperitoneum and/or mesenteric region [1]. Lastly, the bacterial theory poses that gas-producing bacteria may cause gas cysts by invading the intraluminal compartments near blood vessels, particularly following a reduction in gas after treatment with antimicrobial drugs [1]. Thus, the pathogenesis of PI is multifactorial, frequently involving underlying GI, pulmonary, or systemic diseases with varied etiologies and prognoses. Pathologically, PI can be further classified by factors such as bowel necrosis, mucosal disruption, increased mucosal permeability, and pulmonary disease [2]. A study was performed including 123 PI patients demonstrating other causes of PI including mesenteric vascular ischemia (35.0%), bowel obstruction (13.8%), chemotherapy (10.6%), adynamic ileus (7.3%), post-anastomosis (3.3%), chronic obstructive pulmonary disease (2.4%), and nonspecific enteritis (2.4%) [2]. 

Hepatic encephalopathy, a neurological manifestation resulting from liver insufficiency, is primarily driven by elevated ammonia levels [3]. The standard treatment for hepatic encephalopathy includes lactulose, often in conjunction with rifaximin. Lactulose, a nonabsorbable disaccharide, functions as a laxative, facilitating the removal of nitrogen-containing substances from the GI tract while inhibiting the growth of ammonia-producing bacteria, thereby lowering ammonia levels [3]. Research indicates significant symptomatic improvement in hepatic encephalopathy with lactulose in 70 to 80% of patients [4]. However, side effects such as severe diarrhea, electrolyte abnormalities, bloating, and vomiting can negatively impact patient compliance [3].

The following case describes a critically ill 53-year-old male who developed severe PI within hours of receiving lactulose. This case underscores the potential for life-threatening side effects of lactulose in patients with advanced hepatic disease and other comorbid conditions.

## Case presentation

A 53-year-old male resident of a long-term care facility with a significant medical history, including esophageal varices and ascites secondary to alcoholic cirrhosis, type 2 diabetes mellitus, schizophrenia, hypothyroidism, gastroesophageal reflux disease (GERD), urinary tract infections (UTIs), pancytopenia, and acute kidney injury (AKI) requiring hemodialysis (scheduled thrice-weekly), presented to the emergency department (ED) with altered mental status and unresponsiveness. 

Prior to his arrival, the patient had received several doses of lactulose at the care facility due to concerns regarding hepatic encephalopathy, and there were no other reported changes to medications at the facility. The patient was last at baseline two days previously and found unresponsive overnight. Initial laboratory results in the ED revealed markedly elevated ammonia levels of 314 µg/dL. The patient was hemodynamically unstable, presenting with a heart rate of 107 beats per minute, blood pressure of 156/83 mmHg, respiratory rate of 23 breaths per minute, and an oxygen saturation level of 95% while receiving 2 liters of supplemental oxygen via nasal cannula. On hospital Day 1, the patient was administered 40 grams of lactulose, resulting in a reduction of ammonia levels to 209 µg/dL four hours later. He was subsequently admitted to the intensive care unit (ICU) for aggressive management of hyperammonemia, likely attributable to hepatic encephalopathy.

**Figure 1:**
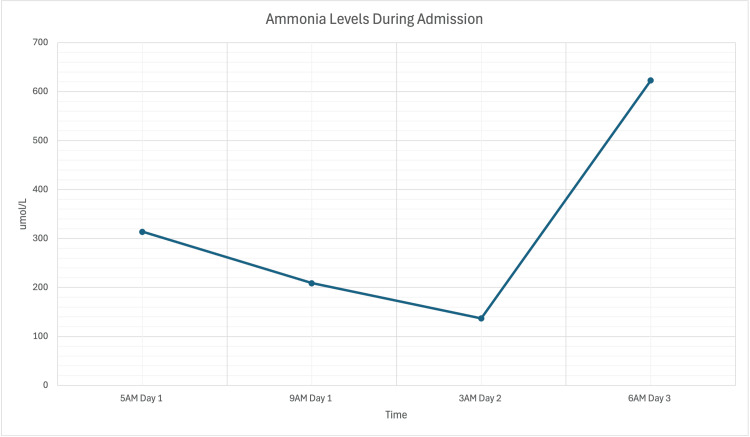
Ammonia levels during admission Reference Range: 16 - 60 umol/L

Gastroenterology was consulted and recommended administering lactulose 30 grams via nasogastric tube (NGT) every two hours until a laxative effect was achieved, after which the regimen could be adjusted to every six to eight hours. Additionally, rifaximin 550 mg via NGT twice daily (BID) was prescribed to address concerns regarding hepatic encephalopathy. The patient was also initiated on ceftriaxone for suspected spontaneous bacterial peritonitis (SBP) in light of fever and acute respiratory distress that necessitated intubation in the ED. Lactulose was adjusted to 30 grams at 5 PM on Day 1 and continued every two hours until 5 PM the following day (Figure 1).

**Figure 2:**
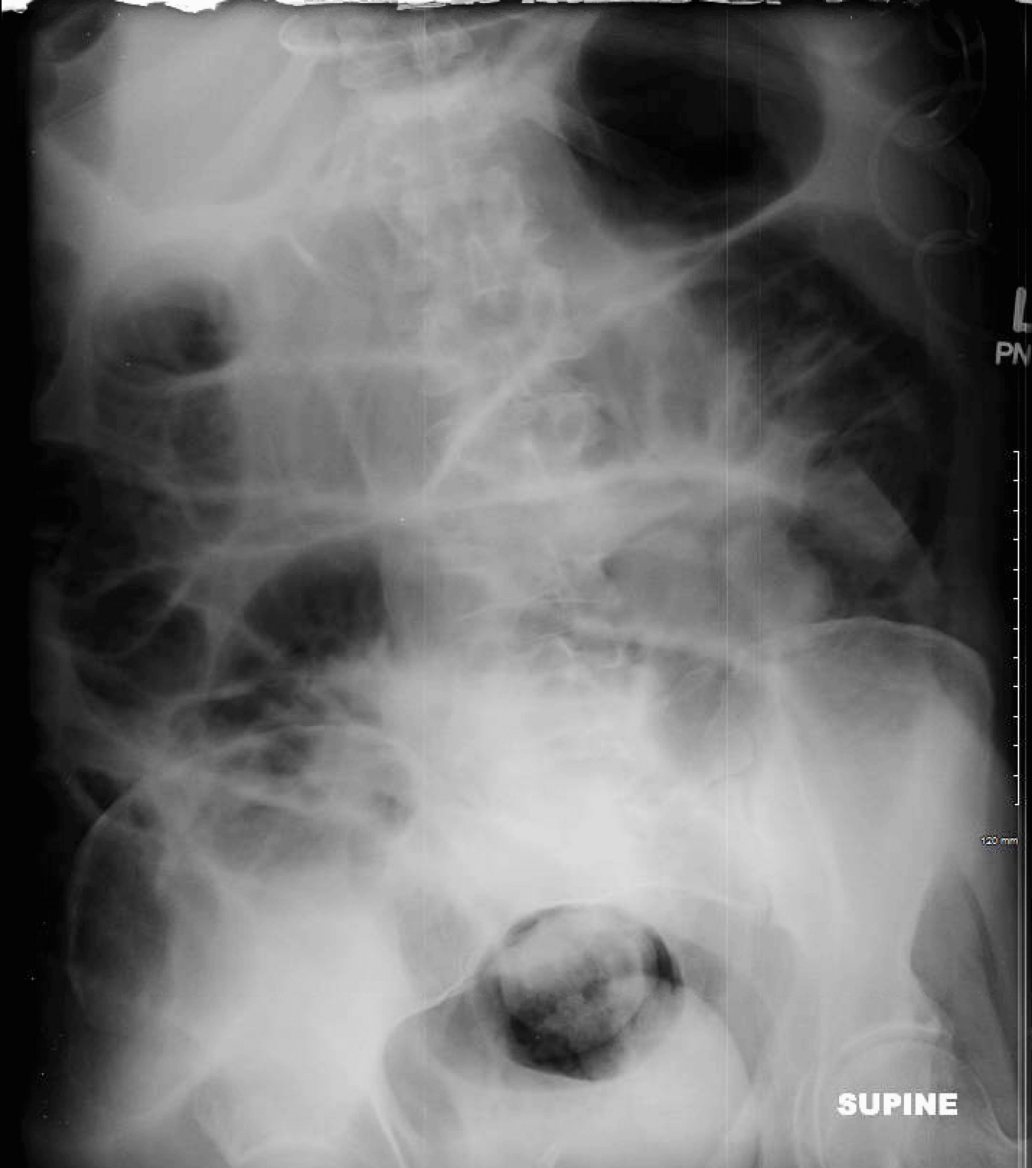
Abdominal X-Ray Coronal View demonstrating scattered air-fluid levels with dilation of the small bowel

Given the patient’s severe shortness of breath and abdominal distension upon admission, he was scheduled for weekly paracentesis. An abdominal X-ray was then performed and demonstrated scattered air-fluid levels with dilation of the small bowel consistent with small bowel obstruction (Figure 2). Subsequently, lactulose and rifaximin were discontinued due to suspected small bowel obstruction versus paralytic ileus. 

**Figure 3:**
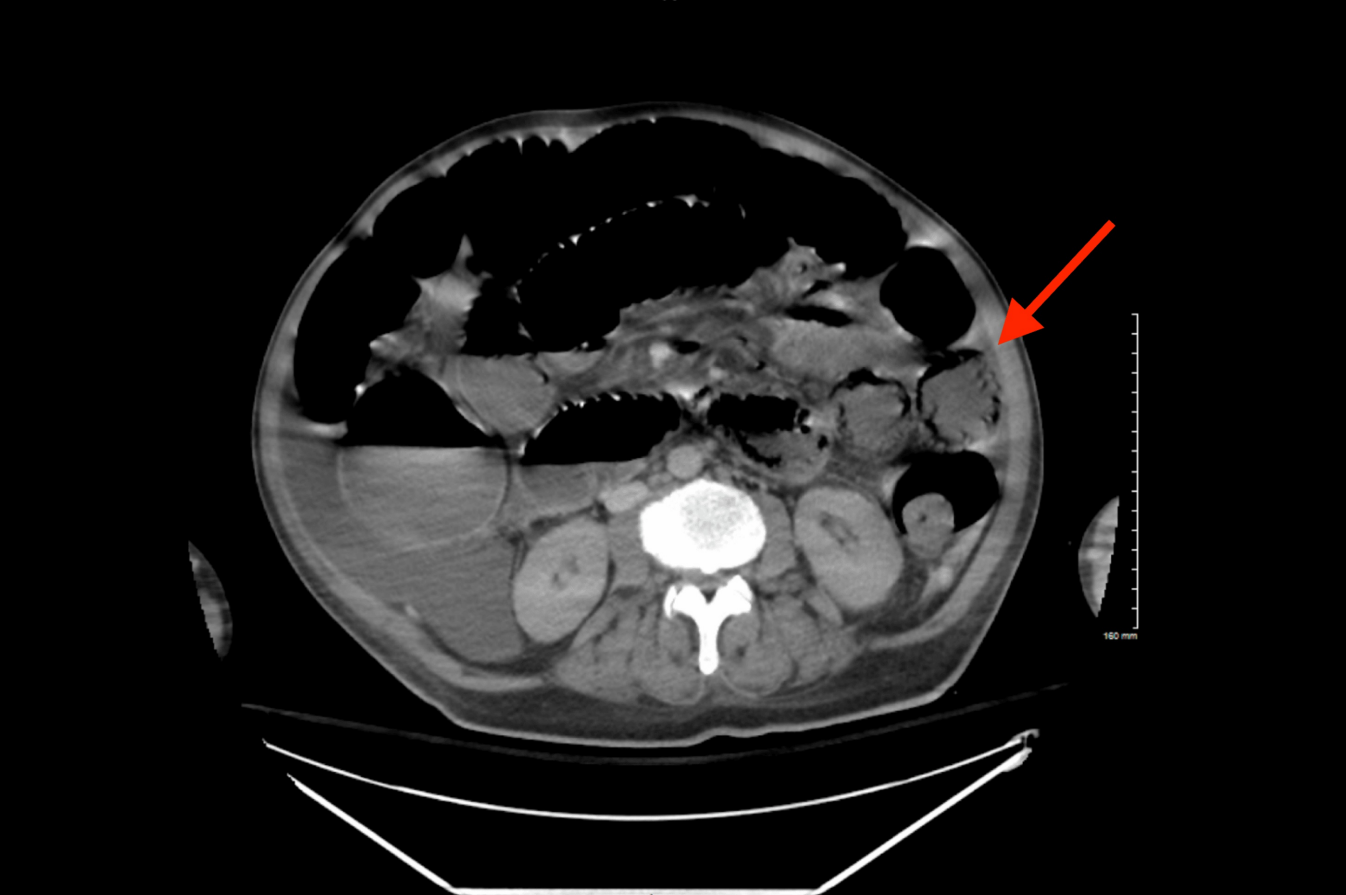
CT abdomen and pelvis with IV contrast axial view demonstrating pneumatosis involving multiple loops of the mid and distal small bowel (red arrow)

Surgical consultation was obtained due to abdominal X-ray abnormalities, leading to the recommendation for an abdominal and pelvis computed tomography (CT) scan. The abdomen and pelvis CT findings demonstrated pneumatosis involving multiple loops of the mid and distal small bowel, consistent with ischemia (Figure 3). A surgical evaluation concluded that the extent of the intestinal ischemia was severe, rendering the affected segments non-salvageable. INR of 1.3, total bilirubin of 2.9, and creatinine of 5.6 were used to calculate the Model for End-Stage Liver Disease (MELD) score. Given the poor prognosis indicated by a MELD score of 23, the decision was made to transition the patient to palliative care.

## Discussion

PI is a complex, multifaceted condition, and its variable prognosis can vary significantly based on the underlying etiology. This case presents a 53-year-old patient with severe hepatic disease and ascites secondary to cirrhosis, who experienced rapid deterioration following lactulose treatment for hepatic encephalopathy.

PI is initially managed conservatively with observation, hyperbaric oxygen therapy, antibiotics, and endoscopy [1]. Surgical intervention is recommended when clinical signs of deterioration are noted, including elevated white blood cell count, imaging demonstrating portal venous gas, and signs of sepsis or acidosis [1]. When evaluating the patient presented above, the MELD score was deemed moderately severe and, therefore, concluded to be inoperable because of the extent of ischemia and severity of the disease. Additionally, lactulose was deemed the cause of PI as no concerns were associated with other interventions including ceftriaxone and rifaximin, and was the only change in medication at an outside long-term care facility. 

This case underscores a potential rare adverse effect of lactulose, especially its association with the induction of PI inpatients with advanced liver disease. Although the exact mechanism linking lactulose to PI remains poorly understood, it may relate to the minimal absorption of lactulose by the gastrointestinal tract [5], leading to the accumulation of gas in the extraluminal space. Commonly reported side effects of lactulose are gastrointestinal in nature, including increased bowel sounds, abdominal bloating, and diarrhea [3, 5]. These side effects raise concerns about potential complications in the gastrointestinal tract.

A study involving 137 patients on long-term lactulose treatment reported that 75% of patients experienced recurrences of hepatic encephalopathy, with 38% of patients becoming noncompliant due to significant gastrointestinal effects [3]. Consequently, the study recommended considering a switch to rifaximin monotherapy and called for further investigation into the adverse effects of lactulose in this population [3].

Additionally, a case report by Goodman and Riley described a patient who developed both PI and pneumoperitoneum following lactulose administration [6]. Notably, the discontinuation of lactulose led to the reversal of PI and the resolution of the patient’s symptoms [6], indicating that lactulose was the primary causative factor. Similarly, Varelas et al. reported a series of cases involving idiopathic PI secondary to lactulose use in patients with cirrhosis [7]. This study reported six of nine patients experiencing the reversibility and resolution of PI upon lactulose discontinuation [7], also suggesting lactulose-induced PI.

In contrast, the PI observed in the 53-year-old patient appeared to be multifactorial, exacerbated by both the underlying advanced hepatic disease and potentially the use of lactulose. Unlike the cases highlighted by Goodman and Riley [6] and Varelas et al. [7], where cessation of lactulose resulted in clinical improvement, this patient’s condition was more severe and ultimately non-salvageable, necessitating a transition to palliative care. This distinction highlights the critical importance of considering the broader clinical context, particularly the severity of the underlying disease when evaluating and managing PI.

## Conclusions

This case exemplifies the complexities involved in managing PI in patients with advanced hepatic disease and multiple comorbidities. This case identified lactulose as the primary cause of PI, as it was the only recent change in the patient's medication regimen reported by the long-term care facility at the time of hospital presentation. The presentation of PI in patients often necessitates a delicate balance between aggressive intervention and compassionate palliative care. Treatment options range from conservative approaches, including antibiotics and observation, to more invasive measures such as surgical intervention. Early recognition and appropriate management are essential to optimize patient outcomes. However, in cases of diffuse PI with a poor prognosis, as in the present case, transitioning to palliative care may be the most appropriate course of action. This underscores the need to reassess the risks and benefits of lactulose in such complex cases and explore alternative therapies when appropriate. Lactulose-induced PI could be a possible side effect seen in advanced hepatic disease and needs to be further studied in order to be better understood.
